# Mitochondrial Modulation by Epigallocatechin 3-Gallate Ameliorates Cisplatin Induced Renal Injury through Decreasing Oxidative/Nitrative Stress, Inflammation and NF-kB in Mice

**DOI:** 10.1371/journal.pone.0124775

**Published:** 2015-04-15

**Authors:** Hao Pan, Jun Chen, Kezhen Shen, Xueping Wang, Ping Wang, Guanghou Fu, Hongzhou Meng, Yimin Wang, Baiye Jin

**Affiliations:** 1 Department of Urology, the First Affiliated Hospital, College of Medicine, Zhejiang University, Hangzhou, Zhejiang, China; 2 Department of Urology, the First People’s Hospital of Wenling City, Wenling, Zhejiang Province, China; 3 Key Laboratory of Combined Multi-organ Transplantation, Ministry of Public Health, the First Affiliated Hospital, College of Medicine, Zhejiang University, Hangzhou, Zhejiang Province, China; University of Kentucky, UNITED STATES

## Abstract

Cancer chemotherapy drug cisplatin is known for its nephrotoxicity. The aim of this study is to investigate whether Epigallocatechin 3-Gallate (EGCG) can reduce cisplatin mediated side effect in kidney and to understand its mechanism of protection against tissue injury. We used a well-established 3-day cisplatin induced nephrotoxicity mice model where EGCG were administered. EGCG is a major active compound in Green Tea and have strong anti-oxidant and anti-inflammatory properties. EGCG protected against cisplatin induced renal dysfunction as measured by serum creatinine and blood urea nitrogen (BUN). EGCG improved cisplatin induced kidney structural damages such as tubular dilatation, cast formation, granulovaculoar degeneration and tubular cell necrosis as evident by PAS staining. Cisplatin induced kidney specific mitochondrial oxidative stress, impaired activities of mitochondrial electron transport chain enzyme complexes, impaired anti-oxidant defense enzyme activities such as glutathione peroxidase (GPX) and manganese superoxide dismutase (MnSOD) in mitochondria, inflammation (tumor necrosis factor α and interleukin 1β), increased accumulation of NF-κB in nuclear fraction, p53 induction, and apoptotic cell death (caspase 3 activity and DNA fragmentation). Treatment of mice with EGCG markedly attenuated cisplatin induced mitochondrial oxidative/nitrative stress, mitochondrial damages to electron transport chain activities and antioxidant defense enzyme activities in mitochondria. These mitochondrial modulations by EGCG led to protection mechanism against cisplatin induced inflammation and apoptotic cell death in mice kidney. As a result, EGCG improved renal function in cisplatin mediated kidney damage. In addition to that, EGCG attenuated cisplatin induced apoptotic cell death and mitochondrial reactive oxygen species (ROS) generation in human kidney tubular cell line HK-2. Thus, our data suggest that EGCG may represent new promising adjunct candidate for cisplatin.

## Introduction

Cisplatin is a highly prescribed drug for cancer. Cisplatin cause cell death to cancer cells after binding to DNA. However, its chemotherapeutic efficacy is severely limited by its nephrotoxicity. The toxicity is mainly due to high amount of cisplatin accumulation in the kidneys with involvement of a specific renal transport systems [1]. Production of reactive oxygen species (ROS) by cisplatin in kidney is crucial to the progression of nephrotoxicity [1,2]. The major sources of ROS production in cisplatin nephrotoxicity are mitochondria and nicotinamide adenine dinucleotide phosphate (NADPH) oxidases [3,4].

Epigallocatechin-3-gallate (EGCG) is a type of catechin and most abundant in Green Tea (*Camellia sinensis Theaceae*). Green Tea has many health benefits such as cancer, inflammation, neuro-protection, cardiovascular disease etc. and is traditionally used as medicine in China and India [5]. Several studies on the beneficial effect of active compound EGCG of Green Tea is mostly focused in cancer [6–8].

Several plant derived phenolic compounds such as Broccoli derived sulforaphane, asian spice derived curcumin, capsaicin, rosemary derived Carnosic acid and arabic gum prevents cisplatin induced nephropathy [9–16]. Transcriptional regulators NF-κB, p53 and NRF2 play important roles as protection mechanism [4,17,18]. Recently, we have demonstrated that metalloporphyrin protects against cisplatin induced renal injury in mice by reducing peroxynitrite formation [19]. Mitochondria targeted antioxidants and PARP pathway inhibitors also demonstrate protection against cisplatin nephrotoxicity in mice [3,20]. One of the key component of cisplatin nephrotoxicity is inflammation followed by recruitment of neutrophils/other leukocytes [4,9,20]. Thus, most cell culture based in vitro studies lack this important component (renal circulation) and limit the understanding of mechanistic aspect.

EGCG mediated protection against cisplatin nephrotoxicity has been reported in rat and mouse model [21,22]. In rat model, Nrf2/HO-1 signaling pathway plays role in protection mechanism by EGCG [22]. In a recent study on mouse model of cisplatin nephrotoxicity, EGCG ameliorate cisplatin induced apoptosis by modulating death receptor Fas, Bax and Bcl-2, those factors involved in extrinsic pathway of apoptosis [21]. However, little is known about detail upstream events of apoptosis which leads to renal protection by EGCG.

In this study we aimed to understand the mechanism of protection by EGCG against cisplatin administration in mice. We used a well-studied 3-day cisplatin induced kidney injury model. EGCG attenuated cisplatin induced serum BUN and creatinine by modulating apoptotic cell death. The mechanism of preventing apoptotic cell death of EGCG was mediated by specific mitochondria mediated protection. EGCG ameliorated cisplatin induced mitochondrial oxidative/nitrative stress and improved cisplatin induced damages of mitochondrial electron transport chain complexes. EGCG also improved functions of anti-oxidant defense enzymes glutathione peroxidase and manganese superoxide dismutase, which lost activity with cisplatin administration in mouse kidney. Thus, EGCG regulated the apoptotic trigger by these combined mitochondrial modulation and also reduced cisplatin induced inflammatory response. In a human tubular cell line HK-2, EGCG reduced cisplatin-induced mitochondrial ROS production and apoptotic cell death.

## Materials and Methods

### Ethics Statement of the study

This study was performed under “Guide for the Care and Use of Laboratory Animals” of the National Institutes of Health. All protocols were approved by the Committee on the Ethics of Animal Experiments of First Affiliated Hospital, College of Medicine, Zhejiang University (Permit Number: 09–028) at the Chinese Academy of Sciences. All efforts were made to minimize suffering.

### Animal Experiments

The mouse strain C57BL/6 was used. Male mice of ~8 weeks age with weights of 18–22 g were used in all experiments. Each experimental group was composed of 6–8 mice. Animals were kept under constant temperature (25°C) and humidity and had access to food and water *ad libitum* throughout the study. Mice were sacrificed under deep anesthesia with 5% isoflurane followed by cervical dislocation on third day (72 hours) after a single injection of cisplatin (cis-diammine platinum (II) dichloride, Sigma) at dose 20 mg/kg i.p. in DMSO/saline vehicle. EGCG was purchased from Sigma Chemical. Drug was dissolved in saline and administered at 100 mg/kg (or as described in text), i.p, for two days, starting 2h before the cisplatin administration. EGCG and vehicle were administered alone (without cisplatin treatment) as separate group.

### Cell culture, treatment and flow cytometry

HK-2 cells (ATCC, VA, USA) were cultured in a Keratinocyte Serum Free Medium and supplements (Life Technologies Inc, CA, USA). Cells were grown under conditions of 95% air and 5% CO2 at 37°C. HK-2 cells were seeded onto 12 well cell culture plates. Cells were treated with a final concentration of ECGC at 10 μM. Following a 2h pretreatment with vehicle (saline) or ECGC, cells were exposed to cisplatin at concentration 60 μM for 24h. Cells were incubated with MitoSOX red at 5μM for 15 mins. Cells were analyzed for simultaneous detection of apoptosis and mitochondrial ROS generation as described earlier in details [23,24]. Flow cytometry was performed with FACS Calibur flow cytometer (BD Biosciences, CA, USA). Early apoptotic cell death was characterized with Annexin V-Allophycocyanin (APC) staining and late apoptosis were determined with Sytox Green staining. Mitochondrial ROS was measured with MitoSOX Red dye and mean intensity was calculated for each samples. Total Cell death was determined for each samples by adding early and late apoptotic cell numbers (Quadrant Q3 and Q2).

### Measurements of serum Creatinine and BUN

On the day of the sacrifice, blood was immediately collected by cardiac puncture and processed. Serum levels of blood urea nitrogen (BUN) and creatinine were measured as described earlier [19].

### Histological evaluation of kidney damage

Kidneys were fixed with 10% formalin for 24 hours in a shaker and embedded in paraffin. Kidneys were sectioned with a microtome, deparaffinized and stained with periodic acid–Schiff (PAS) reagents for histological examination as described earlier [19]. Tubular damage in PAS-stained sections was examined under the microscope and scored based on the percentage of cortical tubules showing epithelial damage: 0-normal; 1<10%; 2–10–25%; 3–26–75%; 4>75%. In details, scoring was carried out based on tubular dilatation, cast formation, granulovaculoar degeneration and tubular cell necrosis. The morphometric examinations were performed in a blinded manner.

### Isolation of Mitochondria from fresh tissue

All manipulations were performed at 4°C or on ice to minimize mitochondrial-membrane and protein degradation. Whole kidney from experimental animals were harvested and immersed in isotonic homogenization buffer (225 mM mannitol/75 mM sucrose/10 mM MOPS/1 mM EGTA/0.5% BSA, pH 7.2). Mitochondria were isolated by differential centrifugation as described earlier [25] In briefly, kidney tissues were homogenized isotonic solution described above. The homogenate was centrifuged at1500g for 5min at 4°C. The supernatant was centrifuged again at 8000g for 15mins at 4°C and the step was repeated one more time. After final centrifugation, mitochondrial pellets were suspended in 150–600 μl of RIPA buffer (Sigma, Hong Kong, China), and protein concentration of mitochondrial fraction lysate was determined by using Bio Rad protein assay kit (BioRad Laboratories, Shanghai, China). The values obtained were corrected for BSA

### Isolation of nuclear fraction from tissue

Nuclear fractions were prepared using commercial nuclear fractionation kit (Pierce, IL,USA) and followed manufacturer’s instruction.

### Estimation of mitochondrial respiratory enzyme activities

The mitochondrial respiratory enzyme activities such as NADH dehydrogenase (Complex I Enzyme Activity Microplate Assay Kit, Abcam Trading Company Ltd. Shanghai, China.), succinate dehydrogenase (Complex II Enzyme Activity Microplate Assay Kit, Abcam Trading Company Ltd. Shanghai, China) Enzyme Activity Microplate Assay Kit, Abcam Trading Company Ltd. Shanghai, China) and cytochrome oxidase (cytochrome c oxidase assay kit, Sigma-Aldrich Co, St Louis, MO, USA) were estimated according to manufacturer’s instruction.

### Quantitative measurement of renal mitochondrial protein nitration in mice

Nitrotyrosine content was evaluated by ELISA. Briefly, an identical amount of protein from mitochondrial fraction was applied to a Maxisorp ELISA plate together with nitrated BSA (Bovine serum albumin) standard and allowed to bind overnight at 4°C. After blocking, wells were incubated at 37°C for 2 h with a mouse monoclonal antibody anti-nitrotyrosine (Upstate Biotechnology, Lake Placid) and then for 1h at 37°C with a peroxidase conjugated goat anti-mouse IgG secondary antibody. After washing, peroxidase reaction product was generated using 3, 3’, 5 5’-tetramethylbenzidine (TMB) peroxidase substrate.

### Quantitative measurement of renal mitochondrial HNE protein adducts

HNE adducts from mitochondrial fraction were determined using OxiSelect™ HNE Adduct ELISA Kit according to manufacturer’s instruction (Cell Biolabs, Genetimes Technology, Inc, Shanghai, China).

### Quantitative determination of MnSOD activity

MnSOD activity was determined from mitochondrial fraction using SOD activity kit (Enzo Life Sciences International, Inc., Plymouth Meeting, PA, USA). This colorimetric assay evaluates the ability of SOD to reduce the superoxide ion concentration generated from the conversion of xanthine and oxygen to uric acid and hydrogen peroxide by xanthine oxidase. SOD activity was determined from percent inhibition of the rate of WST-1-formazan formation with readout at 450 nm. Each sample was loaded in a 96 well plate to the final amount of 10μg/well. A kinetic assay was performed according to manufacturer’s instruction. SOD activity was expressed as fold change compared to vehicle control.

### Quantitative Glutathione Peroxidase Assay from mitochondrial fraction

Mitochondrial glutathione peroxidase was measured using Glutathione Peroxidase (GPX) Assay Kit (Abcam Trading Company Ltd. Shanghai, China) according to manufacturer’s instruction. Glutathione peroxidase reduces cumene hydroperoxide while oxidizing GSH to GSSG. The generated GSSG is reduced to GSH with consumption of NADPH by GR. The decrease of NADPH (measured at 340 nm) is proportional to GPX activity. The GPX activity was displayed as fold change compared to vehicle.

### Quantitative measurement of renal apoptosis in mice

Caspase-3/7 activity of the lysate was measured using Apo-One Homogenous caspase-3/7 Assay Kit (Promega Corp., Madison, WI, USA). An aliquot of 100μl caspase assay agent was added to equal volume tissue lysate in each well, mixed on a plate shaker for 2 h at 37°C in dark and the fluorescence was measured [19].

### Quantitative measurement of renal DNA fragmentation in mice

The DNA fragmentation assay was carried out in tissue extracts using a commercially available kit (Cell Death Detection ELISA Kit, Roche China Ltd., Shanghai) according to manufacturer’s instructions [19].

### Real-time PCR

Isolation of RNA and Real-time PCR were carried out as described earlier [19]. The primer sets for TNFα, IL1β and β-actin were purchased from Qiagen (Pudong, Shanghai, China).

### Statistical Analysis

All data were presented as the means ± SEMs. Multiple comparisons (Tukeys) were performed using one way ANOVA. The analyses were performed with Graph Pad Prism software Version 6.01 (GraphPad Software, Inc, CA, USA). A p value <0.05 was considered statistically significant.

## Results and Discussions

### Effect of EGCG in cisplatin induced kidney dysfunction and tubular damage

**Fig 1:**
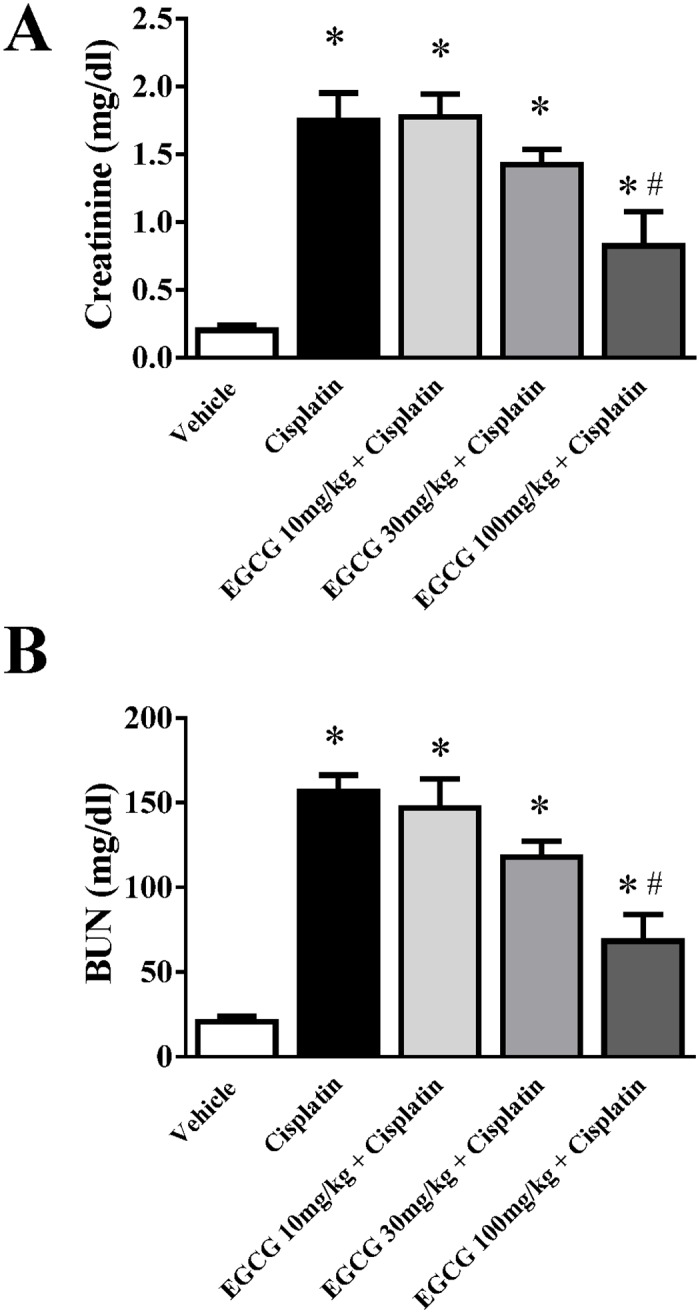
Effect of EGCG on cisplatin-induced renal dysfunction in mice. Cisplatin caused significant renal dysfunction as measured by the levels of creatinine (Panel A) and BUN at 72 hours (Panel B). Cisplatin administration resulted in severe kidney injury which was attenuated by EGCG treatment (at dose 100mg/kg). Results are mean ± S.E.M. n = 4/group.* p<0.05 versus vehicle; and # p<0.05 versus cisplatin.

Cisplatin caused significant renal dysfunction at 72 hours as observed in kidney injury parameters creatinine and BUN (Fig 1). Cisplatin administration resulted 8.75 and 7.6-fold increase in Creatinine and BUN respectively. EGCG attenuated both kidney injury markers in a dose dependent manner. EGCG at 100mg/kg reduced significantly kidney injury as shown by decrease in serum level of BUN (156.5 to 68.2) and creatinine (1.7 to 0.8). EGCG at 100mg/kg (also designated as EGCG 100) was most protective and used for all other experiments described in this study. Dose dependent studies of EGCG in mice demonstrated a linear dose relationship up to 500mg/kg and plateaued between 500 and 2000 mg/kg [26]. Relative bioavailability was between 1.6% at a low dose (75 mg/kg body weight) and 13.9% at higher doses (250 mg/kg and 400 mg/kg) when provided orally to healthy volunteers [27]. EGCG does not have any effect on other organs in mice at dose provided but a single high oral dose of 1500mg/kg in mice resulted hepatotoxicity [28].

**Fig 2:**
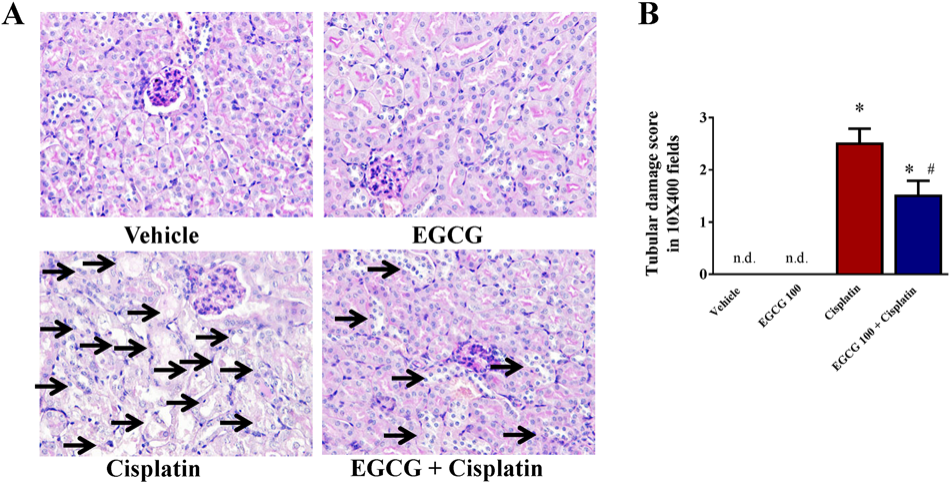
Effect of EGCG on cisplatin-induced kidney tubular damage in mice. Cisplatin administration resulted in severe tubular damage as shown by histological examination using PAS staining. The damage was attenuated by EGCG treatment at dose 100mg/kg (designated as EGCG 100). Results are mean ± S.E.M., n = 4/group.* p<0.05 versus vehicle; and # p<0.05 versus cisplatin.

Cisplatin also induced significant tubular damages in kidney and the damage was examined by PAS staining. Histological examination and quantification revealed necrosis, vacuolation, and desquamation of epithelial cells in the renal tubules indicated by arrow (Fig 2). The damage was significantly attenuated by EGCG 100 treatment in mice. The primary target of cisplatin is tubular epithelial cells as reported earlier [29].

### Effect of EGCG on cisplatin induced mitochondrial oxidative/nitrative stress in kidney

**Fig 3:**
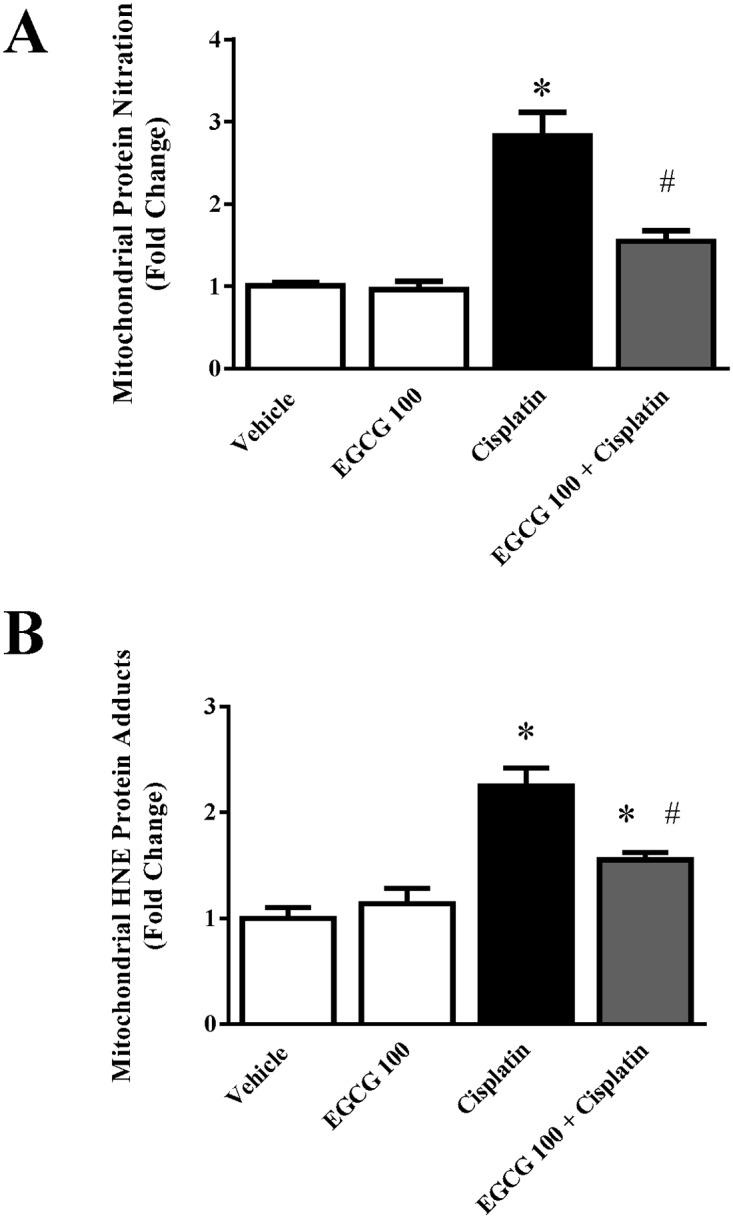
Effect of EGCG on cisplatin-induced oxidative/nitrative stress in mitochondria. Panel A. Quantitative measurement of protein nitration from mitochondrial fraction by ELISA demonstrated cisplatin induced content of protein niration. EGCG attenuated cisplatin induced mitochondrial protein nitration. Panel B. Quantitative measurement of HNE adducts from mitochondrial fraction by ELISA. A trend similar to protein nitration was also observed. Results are mean ± S.E.M. n = 4/group.* p<0.05 versus vehicle; and # p<0.05 versus cisplatin.

It has been reported earlier that EGCG accumulates in mitochondria and modulate oxidative stress [30]. Based on those finding, we examined the effect of EGCG in cisplatin induced mitochondrial oxidative footprints such as HNE protein adducts and protein nitration from isolated mitochondria. Two oxidative/nitrative stress markers HNE and protein nitration were increased 2.2 and 2.8 fold in cisplatin treated mice (Fig 3). Treatment with EGCG reduced mitochondrial oxidative stress significantly (45% and 30% for HNE and nitration respectively). EGCG alone did not produce any significant changes in values. Both oxidative markers are widely used in the literature [31–33]. It is well known that mitochondria are the major source for reactive oxygen species (ROS) generation and the sources include electron transport chain and NOX4 [34,35]. EGCG is a very potent antioxidant and its accumulation in mitochondria might play significant role in reducing mitochondrial oxidative stress [30,36]. We used another marker of oxidative stress which is protein nitration, a product of peroxynitrite formation [33]. Peroxynitrite is formed by superoxide (from mitochondria) and nitric oxide (from cytosolic iNOS) in a diffusion controlled reaction. It might suggest that the ameliorative effect of EGCG on cisplatin induced protein nitration was less pronounced compared to HNE adduct formation due to cytoplasmic component of the reaction. EGCG demonstrated to reduce cisplatin induced mitochondrial oxidative and nitrative stress and thus modulated the trigger of apoptotic cell death. To address this issue further, we examined the role of EGCG on mitochondrial sources of ROS namely electron transport chain enzyme complexes.

### Effect of EGCG on cisplatin induced mitochondrial respiratory enzyme complex activities in kidney

**Fig 4:**
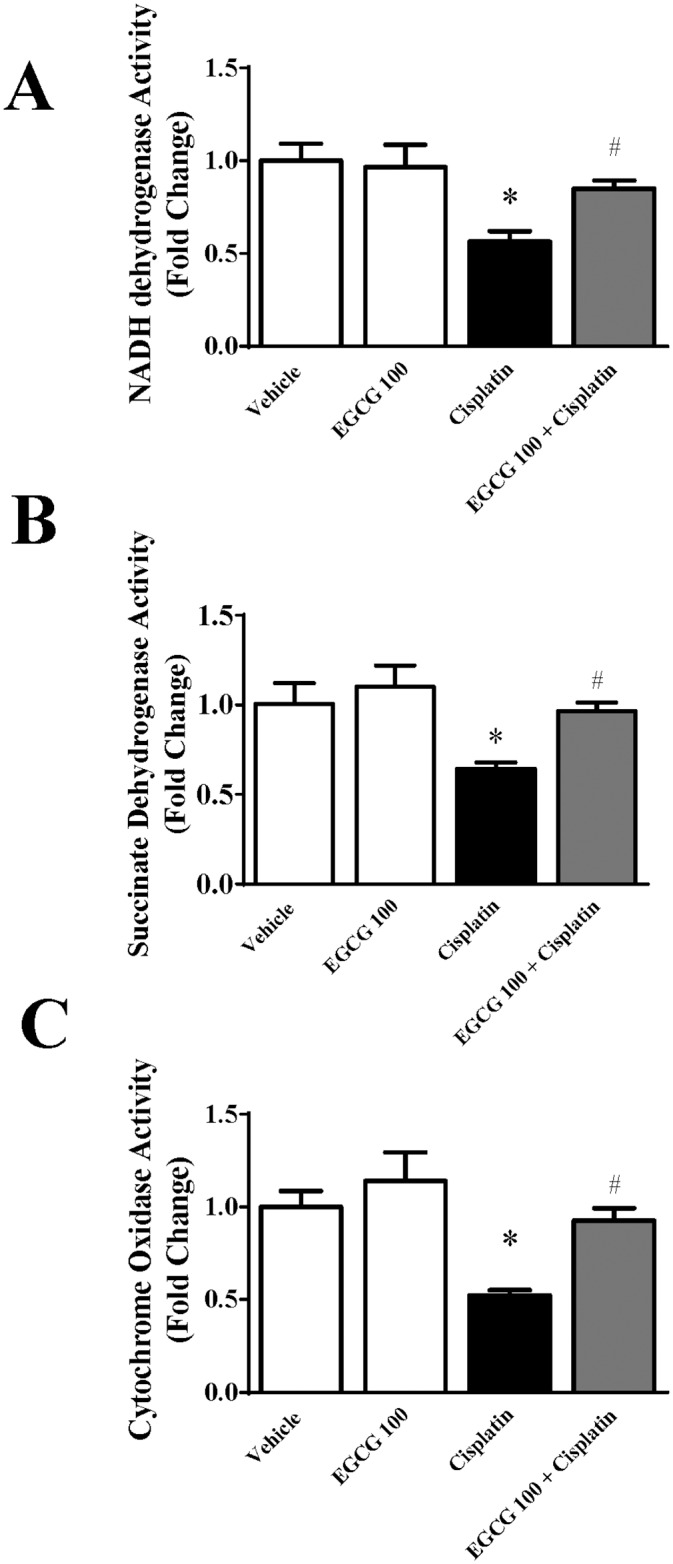
Effect of EGCG on cisplatin-induced changes in mitochondrial enzyme complex activities in mice. Cisplatin reduced the enzyme activities of mitochondrial electron transport chain namely (Panel A) NADH dehydrogenase Activity (Complex I), (Panel B) Succinate Dehydrogenase Activity (Complex II) and (Panel C) Cytochrome Oxidase Activity (Complex IV). EGCG administration protected mitochondrial enzyme against cisplatin induced damages. Results are mean ± S.E.M. n = 4/group.* p<0.05 versus vehicle; and # p<0.05 versus cisplatin.

**Fig 5:**
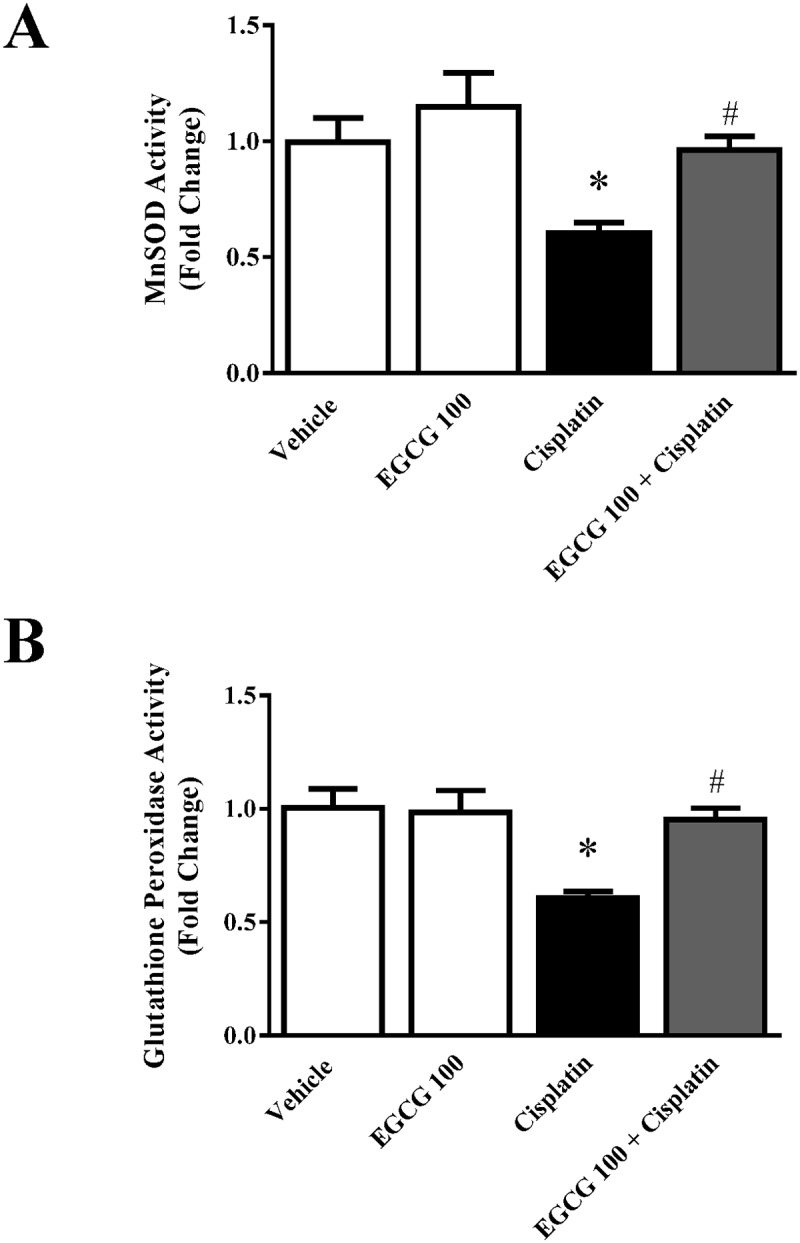
Effect of EGCG on cisplatin-induced nitrative stress in mice. Panel A. Histological examination revealed significant protein nitration in the renal tubules of the cisplatin-treated group. EGCG treatment reduced the level of protein nitration similar to vehicle level. Panel B. Quantitative measurement of 3-nitrotyrosine adducts in protein also demonstrated similar trend. Results are mean ± S.E.M. n = 4/group.* p<0.05 versus vehicle; and # p<0.05 versus cisplatin.

Mitochondrial electron transport chain complexes have been shown to play crucial role in cisplatin induced nephrotoxicity [3,4]. We also examined the effect of EGCG on three active mitochondrial complexes activities namely NADH dehydrogenase Activity (Complex I), Succinate Dehydrogenase Activity (Complex II) and Cytochrome Oxidase Activity (Complex IV). Cisplatin significantly reduced all three enzyme activities namely complex I, complex II and Complex IV to 44%, 36% and 47.5% respectively (Fig 4). Treatment with EGCG at 100mg/kg improved those activities to normal level. EGCG alone did not produce any significant changes. The specific effect of EGCG on mitochondrial complex has two aspects. First, the antioxidant property of EGCG reduce the oxidative stress in mitochondrial from cytosolic ROS sources and thus preventing production of more ROS from mitochondrial enzyme complex sources [4]. The other aspect is the role of EGCG in mitochondrial antioxidant defense mechanisms such as MnSOD and Glutathione peroxidase activity. We also carried out those enzyme activities in mitochondrial fraction from kidney tissue suspension. Indeed, both antioxidant defense enzymes reduced in the presence of cisplatin as reported earlier [30,37]. EGCG restored both activities to normal level (Fig 5). Similar renal mitochondrial protection against oxidative stress was observed in epicatechin, a close family member of EGCG [38]. EGCG improved cisplatin induced damages to mitochondrial electron transport chain complexes in mouse kidney by increasing their activities to normal level.

### Effect of EGCG on cisplatin induced inflammation in kidney

**Fig 6:**
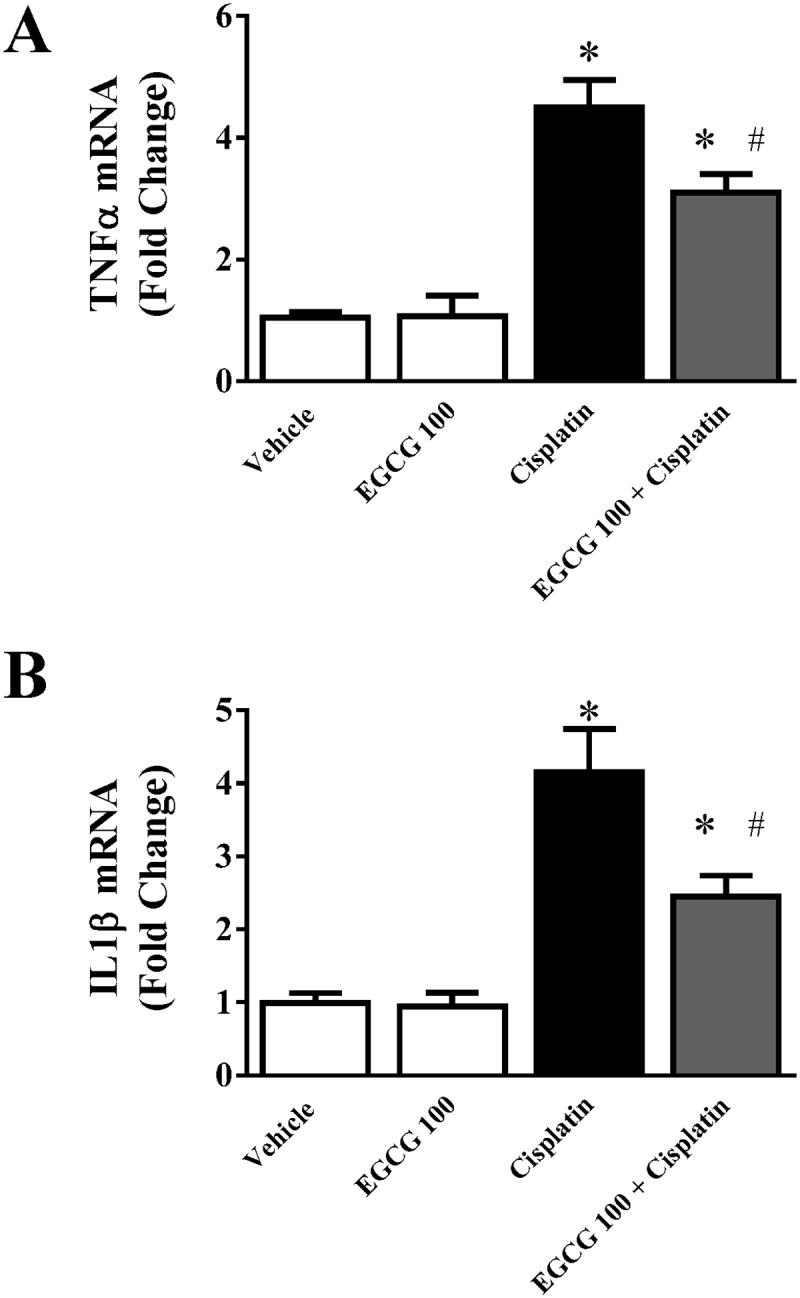
Effect of EGCG on cisplatin-induced pro-inflammatory cytokines in mice. Real-time PCR based analyses of pro-inflammatory cytokines TNFα and IL1β indicated profound increase in cisplatin treated mice. EGCG treatment attenuated cisplatin induced TNFα and IL1β production. Results are mean ± S.E.M. n = 4/group.* p<0.05 versus vehicle; and # p<0.05 versus cisplatin.

Inflammation plays a critical role in cisplatin induced nephrotoxicity [9,39,40]. In order to evaluate the role of EGCG in cisplatin induced renal inflammation, we analyzed two pro-inflammatory cytokines (TNFα and IL1β). As reported earlier, both pro-inflammatory cytokines TNFα and IL1β increased at mRNA level to 4.5 and 4.15 fold respectively in response to cisplatin toxicity. As shown in Fig 6, EGCG attenuates cisplatin induced TNFα and IL1β mRNA to 30% and 41% respectively. EGCG alone did not change those cytokine mRNA level significantly. The inflammatory response followed by infiltration of neutrophil supports its important role and has been reported earlier [4,9,19].TNFα is important in cisplatin mediated toxicity and key player in kidney injury. Studies with TNFα antibody treatment or TNFα knock out mice are resistance to cisplatin-induced nephrotoxicity [40,41]. Although anti-inflammatory role of EGCG has been reported earlier in various immunology models [42–44], this study is first report of its anti-inflammatory role in a drug induced tissue injury model. Anti-inflammatory role of EGCG was reported in a surgical obstructive nephropathy model earlier [45,46]. Here, EGCG demonstrated its anti-inflammatory role in response to cisplatin induced inflammatory process.

### Effect of EGCG on cisplatin induced modulation of transcription factor NFκB and p53 in kidney

**Fig 7:**
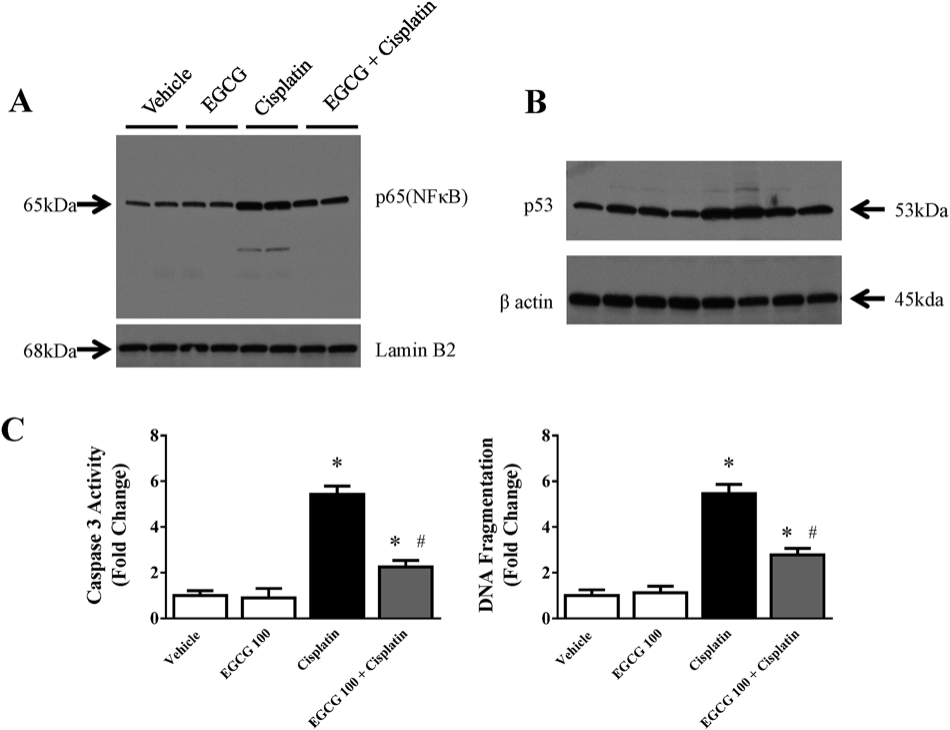
Effect of EGCG on cisplatin-induced nuclear translocation of NFκB, induction of p53 and apoptosis. Panel A. Immuno-blot analyses of nuclear fraction from kidney demonstrated increase in p65 in cisplatin treated mice. The level is reduced in EGCG and cisplatin treated group. Panel B. Immuno-blot analyses of total lysate from kidney demonstrated increase in p53 in cisplatin treated mice. Panel C. Quantitative measurement of Caspase 3 activity and DNA fragmentation. Results are mean ± S.E.M. n = 4/group.* p<0.05 versus vehicle; and # p<0.05 versus cisplatin

NFκB contributes to oxidative stress, inflammation and mitochondrial gene regulation. We examined the effect of EGCG on NF-κB (p65) protein expression in nuclear fraction. Western blot analyses demonstrated significant increase in NF-κB (p65) in nuclear fraction from cisplatin administered mice when compared to vehicle or EGCG treated groups (Fig 7A). EGCG administration significantly decreased the amount of p65 in nuclear fraction compared to cisplatin treated group. In recent study the important role of NF-κB (p65) has been demonstrated in cisplatin induced kidney injury [4]. Another important transcription regulator in cisplatin nephrotoxicity is p53 and is also modulated by EGCG. Cisplatin significantly increase p53 protein level and the induction is attenuated by EGCG treatment (Fig 7B). There is no significant change with EGCG treatment alone. It has been demonstrated earlier that cisplatin induced tubular cell apoptosis and renal failure were ameliorated by p53 inhibitor pifithrin or p53knock out mice [47]. The mechanism of p53 is not clear. However, it is believed that cisplatin upon entering tubular cell, crosslink with DNA in its active form and that cause DNA damage [48]. EGCG either acted directly as antioxidant or indirectly via other mechanisms which reduced p53 induced DNA damage. We demonstrated here that EGCG modulated cisplatin induced NF-κB nuclear accumulation and p53 induction. EGCG might influence both inflammatory response and apoptotic cell death through these transcriptional regulators.

It has been proposed in obstructive nephropathy study that both NF-κB and NRF-2 are induced by EGCG and translocate to nucleus. They promote heme oxygenase 1(HO-1) production and modulate inflammation [46]. We also observed the importance of EGCG induced NF-κB in cisplatin induced nephropathy. Interestingly, Nrf-2/HO-1 pathway is a key antioxidant defense system inside cells which modulates both antioxidant and anti-inflammatory response and they are also linked to NF-κB. Thus, EGCG modulation of NF-κB and Nrf-2 is a critical element for oxidative stress induced cell death and inflammation or both in acute kidney injury. Green tea extract also reduce contrast media induced acute kidney injury in rats [49]. Thus, EGCG is beneficial in a variety of acute kidney injury models.

### Effect of EGCG on cisplatin induced apoptotic cell death in kidney

We also measure DNA fragmentation in our study and EGCG indeed reduced cisplatin induced DNA fragmentation (Fig 7C) to from 5.5 fold increase to 2.8 fold (compared to vehicle control). An early marker of apoptosis, caspase 3/7 activity, also demonstrated EGCG attenuates cisplatin induced apoptosis to 58% (5.4 fold to 2.25 fold, Fig 7D). It is well known that cisplatin leads to apoptosis in mouse kidney tubular cells followed by renal failure and thus increase in serum BUN and creatinine [21,39]. Both DNA fragmentation and caspase 3/7 activity presented here are markers for apoptotic cell death and has been used earlier in cisplatin induced nephrotoxicity [50,51]. Several mechanisms have been proposed for the trigger for apoptotic cell death [52–54]. However, recent studies demonstrated that mitochondria plays a key role in cisplatin induced renal cell death [3,55–57]. In addition to that, mitochondria are functional regulator of apoptosis [58]. In this study, we demonstrated that EGCG targets mitochondria and thus modulate apoptotic cell death.

### Effect of EGCG on cisplatin induced apoptotic cell death and mitochondrial ROS generation in human tubular epithelial cells HK-2

**Fig 8:**
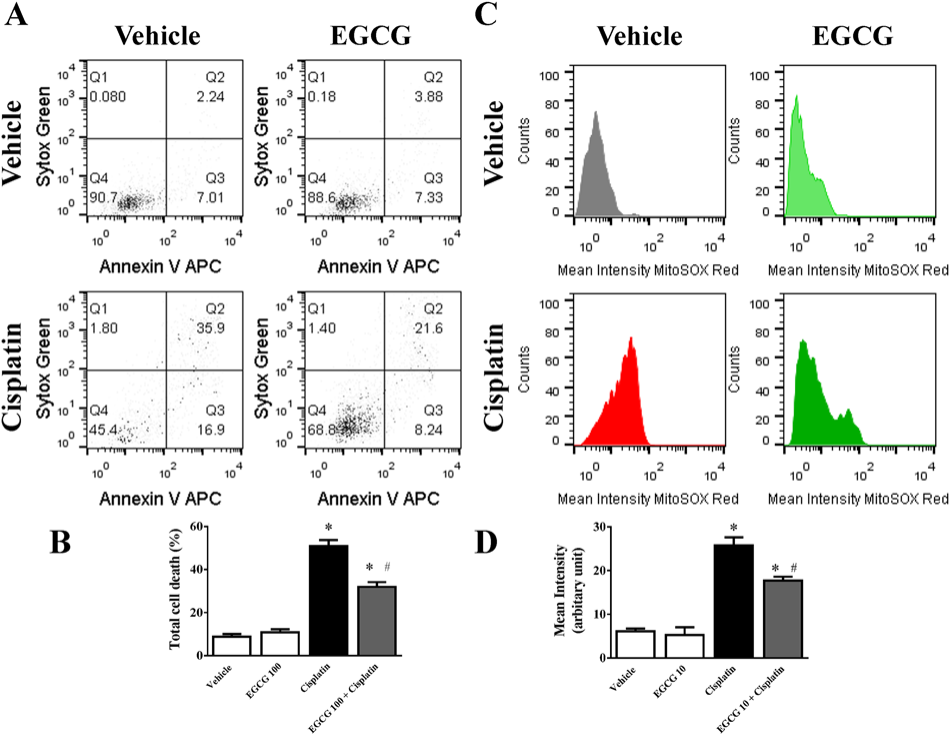
Effect of EGCG on cisplatin-induced apoptosis and mitochondrial ROS generation in HK-2 cells. Panel A: Representative dot plot data showing early apoptosis (AnnexinV-APC staining) and late apoptosis (with Sytox Green) as measured by flow cytometry. Cisplatin was used at 60μM and EGCG was added at 10μM 2h prior to cisplatin addition. Panel B. Quantitative determinations of total cell death including early and late apoptosis were presented. Results are mean ± S.E.M. n = 4/group.* p<0.05 versus vehicle; and # p<0.05 versus cisplatin. Panel C: Representative histogram analyses of MitoSOX Red intensities from the same samples were presented. Panel D: Quantitative determinations of MitoSOX Red mean intensities. Results are mean ± S.E.M. n = 4/group.* p<0.05 versus vehicle; and # p<0.05 versus cisplatin.

Simultaneous detection of apoptotic cell death and mitochondrial ROS production in cisplatin treated (60μM) HK-2 cells for 24 hours results significant cell death (5.8 fold) and increase in mitochondrial ROS production (4.2 fold) as measured by mean intensity of MitoSOX RED using flow cytometry (Fig 8). Treatment with EGCG at10μM reduced both apoptosis (37%) and mitochondrial ROS production (31.3%). These data demonstrated that EGCG contributed mitochondrial ROS mediated protection in cisplatin induced cell death for human kidney tubular cell line. EGCG is implicated in reducing ROS production and mitochondrial integrity earlier in fibroblast [59]. We would like to caution that cell line data might be different compared to cisplatin induced tissue injury situation where the process is more complex. However, EGCG demonstrated direct role by modulating cisplatin induced mitochondrial ROS in live kidney tubular cells.

## Conclusion

**Fig 9:**
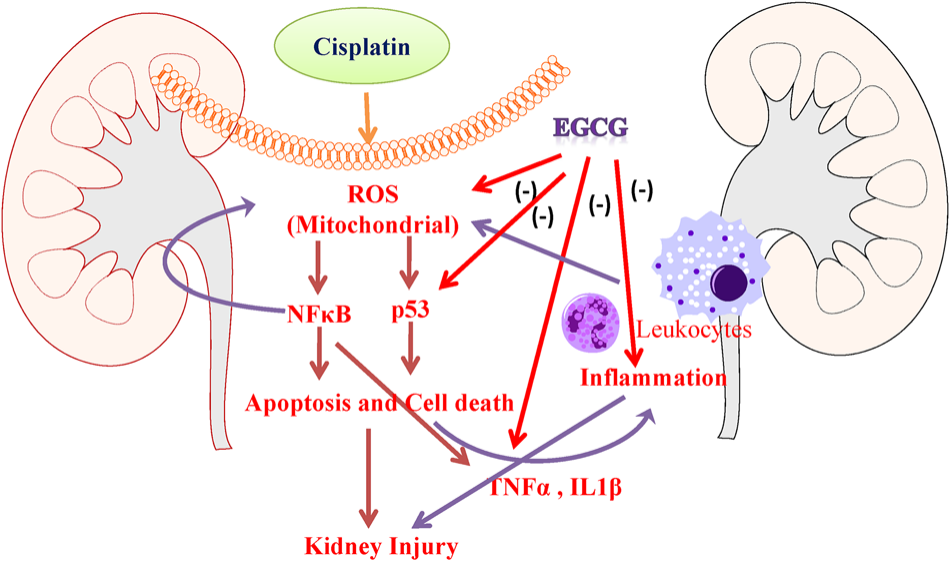
Schematic diagram of protection mechanism of EGCG in cisplatin induced kidney injury. EGCG inhibit cisplatin induced mitochondrial ROS (Reactive Oxygen Species) in the renal tubular cells which caused cell death. Cell death in the tubular cells leads to pro-inflammatory response with cytokines (TNFα and IL1β). These process leads to leukocytes infiltration with additional burst of oxidative stress. EGCG also neutralize these pro-inflammatory cytokines. All combinatorial effects leads to reduced inflammation and cell death, thus protecting against cisplatin induced kidney injury.

Briefly, in this study we demonstrated EGCG protects again cisplatin induced renal injury through mitochondrial protection in three ways (1) by improving cisplatin induced mitochondrial electron chain complexes, (2) by improving mitochondrial antioxidant function in enzymes MnSOD and GPX, (3) improving cisplatin induced mitochondrial oxidative/nitrative damage and (4) anti-inflammatory effect (Fig 9). Here, we also demonstrated that EGCG reduced cisplatin induced inflammatory response by lowering pro-inflammatory cytokines and neutrophil infiltration. EGCG also modulated cisplatin induced nuclear translocation of NFκB (p65), which has role in inflammation and oxidative stress. Another transcription factor p53, DNA damage response factor, also modulated by EGCG in cisplatin affected tubular cells. This modulation lead to improvement of cisplatin induced DNA fragmentation and Caspase activity or apoptosis by EGCG in renal tissue. These protective effects of EGCG, also a natural ingredient of Green Tea, will be very useful for therapeutic purpose in cisplatin induced kidney injury or other kidney injury models where mitochondria/oxidative stress/inflammation is involved. It is important to note that EGCG is reported to be effective against various cancer [60–64] and this will make is ideal adjunct candidate for cisplatin chemotherapy.

## References

[1] Sanchez-Gonzalez PD, Lopez-Hernandez FJ, Lopez-Novoa JM, Morales AI (2011) An integrative view of the pathophysiological events leading to cisplatin nephrotoxicity. Crit Rev Toxicol 41: 803–821. 10.3109/10408444.2011.602662 21838551

[2] Sahu BD, Kuncha M, Sindhura GJ, Sistla R (2013) Hesperidin attenuates cisplatin-induced acute renal injury by decreasing oxidative stress, inflammation and DNA damage. Phytomedicine 20: 453–460. 10.1016/j.phymed.2012.12.001 23353054

[3] Mukhopadhyay P, Horvath B, Zsengeller Z, Zielonka J, Tanchian G, et al (2012) Mitochondrial-targeted antioxidants represent a promising approach for prevention of cisplatin-induced nephropathy. Free Radic Biol Med 52: 497–506. 10.1016/j.freeradbiomed.2011.11.001 22120494 PMC3253235

[4] Sahu BD, Kalvala AK, Koneru M, Mahesh Kumar J, Kuncha M, et al (2014) Ameliorative Effect of Fisetin on Cisplatin-Induced Nephrotoxicity in Rats via Modulation of NF-kappaB Activation and Antioxidant Defence. PLoS One 9: e105070 10.1371/journal.pone.0105070 25184746 PMC4153571

[5] Chacko SM, Thambi PT, Kuttan R, Nishigaki I (2010) Beneficial effects of green tea: a literature review. Chin Med 5: 13 10.1186/1749-8546-5-13 20370896 PMC2855614

[6] Shirakami Y, Shimizu M, Moriwaki H (2012) Cancer chemoprevention with green tea catechins: from bench to bed. Curr Drug Targets 13: 1842–1857. 23140294 10.2174/138945012804545506

[7] Connors SK, Chornokur G, Kumar NB (2012) New insights into the mechanisms of green tea catechins in the chemoprevention of prostate cancer. Nutr Cancer 64: 4–22. 10.1080/01635581.2012.630158 22098273 PMC3665011

[8] Shimizu M, Adachi S, Masuda M, Kozawa O, Moriwaki H (2011) Cancer chemoprevention with green tea catechins by targeting receptor tyrosine kinases. Mol Nutr Food Res 55: 832–843. 10.1002/mnfr.201000622 21538846

[9] Guerrero-Beltran CE, Mukhopadhyay P, Horvath B, Rajesh M, Tapia E, et al (2012) Sulforaphane, a natural constituent of broccoli, prevents cell death and inflammation in nephropathy. J Nutr Biochem 23: 494–500. 10.1016/j.jnutbio.2011.02.004 21684138 PMC3179776

[10] Trujillo J, Chirino YI, Molina-Jijon E, Anderica-Romero AC, Tapia E, et al (2013) Renoprotective effect of the antioxidant curcumin: Recent findings. Redox Biol 1: 448–456. 10.1016/j.redox.2013.09.003 24191240 PMC3814973

[11] Guerrero-Beltran CE, Calderon-Oliver M, Martinez-Abundis E, Tapia E, Zarco-Marquez G, et al (2010) Protective effect of sulforaphane against cisplatin-induced mitochondrial alterations and impairment in the activity of NAD(P)H: quinone oxidoreductase 1 and gamma glutamyl cysteine ligase: studies in mitochondria isolated from rat kidney and in LLC-PK1 cells. Toxicol Lett 199: 80–92. 10.1016/j.toxlet.2010.08.009 20732396

[12] Kursunluoglu G, Kayali HA, Taskiran D (2014) The Effect of Cisplatin Toxicity and Capsaicin on Electron Transport Chain in Liver and Kidney of Sprague Dawley Rats. Cell Biochem Biophys. 10.1007/s12013-014-9857-z 24648159

[13] Jung SH, Kim HJ, Oh GS, Shen A, Lee S, et al (2014) Capsaicin Ameliorates Cisplatin-Induced Renal Injury through Induction of Heme Oxygenase-1. Mol Cells 37: 234–240. 10.14348/molcells.2014.2322 24642709 PMC3969044

[14] Al-Majed AA, Abd-Allah AR, Al-Rikabi AC, Al-Shabanah OA, Mostafa AM (2003) Effect of oral administration of Arabic gum on cisplatin-induced nephrotoxicity in rats. J Biochem Mol Toxicol 17: 146–153. 12815610 10.1002/jbt.10072

[15] Gaona-Gaona L, Molina-Jijon E, Tapia E, Zazueta C, Hernandez-Pando R, et al (2011) Protective effect of sulforaphane pretreatment against cisplatin-induced liver and mitochondrial oxidant damage in rats. Toxicology 286: 20–27. 10.1016/j.tox.2011.04.014 21575670

[16] Sahu BD, Rentam KK, Putcha UK, Kuncha M, Vegi GM, et al (2011) Carnosic acid attenuates renal injury in an experimental model of rat cisplatin-induced nephrotoxicity. Food Chem Toxicol 49: 3090–3097. 10.1016/j.fct.2011.08.018 21930180

[17] Jiang M, Dong Z (2008) Regulation and pathological role of p53 in cisplatin nephrotoxicity. J Pharmacol Exp Ther 327: 300–307. 10.1124/jpet.108.139162 18682572

[18] Kang KP, Park SK, Kim DH, Sung MJ, Jung YJ, et al (2011) Luteolin ameliorates cisplatin-induced acute kidney injury in mice by regulation of p53-dependent renal tubular apoptosis. Nephrol Dial Transplant 26: 814–822. 10.1093/ndt/gfq528 20817674

[19] Pan H, Shen K, Wang X, Meng H, Wang C, et al (2014) Protective effect of metalloporphyrins against cisplatin-induced kidney injury in mice. PLoS One 9: e86057 10.1371/journal.pone.0086057 24454954 PMC3891880

[20] Mukhopadhyay P, Horvath B, Kechrid M, Tanchian G, Rajesh M, et al (2011) Poly(ADP-ribose) polymerase-1 is a key mediator of cisplatin-induced kidney inflammation and injury. Free Radic Biol Med 51: 1774–1788. 10.1016/j.freeradbiomed.2011.08.006 21884784 PMC3207278

[21] Zou P, Song J, Jiang B, Pei F, Chen B, et al (2014) Epigallocatechin-3-gallate protects against cisplatin nephrotoxicity by inhibiting the apoptosis in mouse. Int J Clin Exp Pathol 7: 4607–4616. 25197333 PMC4152023

[22] Sahin K, Tuzcu M, Gencoglu H, Dogukan A, Timurkan M, et al (2010) Epigallocatechin-3-gallate activates Nrf2/HO-1 signaling pathway in cisplatin-induced nephrotoxicity in rats. Life Sci 87: 240–245. 10.1016/j.lfs.2010.06.014 20619277

[23] Mukhopadhyay P, Rajesh M, Hasko G, Hawkins BJ, Madesh M, et al (2007) Simultaneous detection of apoptosis and mitochondrial superoxide production in live cells by flow cytometry and confocal microscopy. Nat Protoc 2: 2295–2301. 17853886 10.1038/nprot.2007.327 PMC2225540

[24] Mukhopadhyay P, Rajesh M, Yoshihiro K, Hasko G, Pacher P (2007) Simple quantitative detection of mitochondrial superoxide production in live cells. Biochem Biophys Res Commun 358: 203–208. 17475217 10.1016/j.bbrc.2007.04.106 PMC2228267

[25] Trounce IA, Kim YL, Jun AS, Wallace DC (1996) Assessment of mitochondrial oxidative phosphorylation in patient muscle biopsies, lymphoblasts, and transmitochondrial cell lines. Methods Enzymol 264: 484–509. 8965721 10.1016/s0076-6879(96)64044-0

[26] Lambert JD, Lee MJ, Diamond L, Ju J, Hong J, et al (2006) Dose-dependent levels of epigallocatechin-3-gallate in human colon cancer cells and mouse plasma and tissues. Drug Metab Dispos 34: 8–11. 16204466 10.1124/dmd.104.003434

[27] Ullmann U, Haller J, Decourt JP, Girault N, Girault J, et al (2003) A single ascending dose study of epigallocatechin gallate in healthy volunteers. J Int Med Res 31: 88–101. 12760312 10.1177/147323000303100205

[28] Lambert JD, Kennett MJ, Sang S, Reuhl KR, Ju J, et al (2010) Hepatotoxicity of high oral dose (-)-epigallocatechin-3-gallate in mice. Food Chem Toxicol 48: 409–416. 10.1016/j.fct.2009.10.030 19883714 PMC2905152

[29] Daugaard G, Abildgaard U, Holstein-Rathlou NH, Amtorp O, Leyssac PP (1987) Effect of cisplatin on renal haemodynamics and tubular function in the dog kidney. Int J Androl 10: 347–351. 3583422 10.1111/j.1365-2605.1987.tb00201.x

[30] Schroeder EK, Kelsey NA, Doyle J, Breed E, Bouchard RJ, et al (2009) Green tea epigallocatechin 3-gallate accumulates in mitochondria and displays a selective antiapoptotic effect against inducers of mitochondrial oxidative stress in neurons. Antioxid Redox Signal 11: 469–480. 10.1089/ARS.2008.2215 18754708 PMC13148728

[31] Suzuki D, Miyata T, Saotome N, Horie K, Inagi R, et al (1999) Immunohistochemical evidence for an increased oxidative stress and carbonyl modification of proteins in diabetic glomerular lesions. J Am Soc Nephrol 10: 822–832. 10203367 10.1681/ASN.V104822

[32] Ceriello A, Mercuri F, Quagliaro L, Assaloni R, Motz E, et al (2001) Detection of nitrotyrosine in the diabetic plasma: evidence of oxidative stress. Diabetologia 44: 834–838. 11508267 10.1007/s001250100529

[33] Pacher P, Beckman JS, Liaudet L (2007) Nitric oxide and peroxynitrite in health and disease. Physiol Rev 87: 315–424. 17237348 10.1152/physrev.00029.2006 PMC2248324

[34] Kuroda J, Ago T, Matsushima S, Zhai P, Schneider MD, et al (2010) NADPH oxidase 4 (Nox4) is a major source of oxidative stress in the failing heart. Proc Natl Acad Sci U S A 107: 15565–15570. 10.1073/pnas.1002178107 20713697 PMC2932625

[35] Murphy MP (2009) How mitochondria produce reactive oxygen species. Biochem J 417: 1–13. 10.1042/BJ20081386 19061483 PMC2605959

[36] Singh BN, Shankar S, Srivastava RK (2011) Green tea catechin, epigallocatechin-3-gallate (EGCG): mechanisms, perspectives and clinical applications. Biochem Pharmacol 82: 1807–1821. 10.1016/j.bcp.2011.07.093 21827739 PMC4082721

[37] Davis CA, Nick HS, Agarwal A (2001) Manganese superoxide dismutase attenuates Cisplatin-induced renal injury: importance of superoxide. J Am Soc Nephrol 12: 2683–2690. 11729237 10.1681/ASN.V12122683

[38] Tanabe K, Tamura Y, Lanaspa MA, Miyazaki M, Suzuki N, et al (2012) Epicatechin limits renal injury by mitochondrial protection in cisplatin nephropathy. Am J Physiol Renal Physiol 303: F1264–1274. 10.1152/ajprenal.00227.2012 22933302 PMC5243204

[39] Pabla N, Dong Z (2008) Cisplatin nephrotoxicity: mechanisms and renoprotective strategies. Kidney Int 73: 994–1007. 10.1038/sj.ki.5002786 18272962

[40] Ramesh G, Reeves WB (2002) TNF-alpha mediates chemokine and cytokine expression and renal injury in cisplatin nephrotoxicity. J Clin Invest 110: 835–842. 12235115 10.1172/JCI15606 PMC151130

[41] Zhang B, Ramesh G, Norbury CC, Reeves WB (2007) Cisplatin-induced nephrotoxicity is mediated by tumor necrosis factor-alpha produced by renal parenchymal cells. Kidney Int 72: 37–44. 17396112 10.1038/sj.ki.5002242

[42] Peairs A, Dai R, Gan L, Shimp S, Rylander MN, et al (2010) Epigallocatechin-3-gallate (EGCG) attenuates inflammation in MRL/lpr mouse mesangial cells. Cell Mol Immunol 7: 123–132. 10.1038/cmi.2010.1 20140007 PMC4001894

[43] Zhong Y, Chiou YS, Pan MH, Shahidi F (2012) Anti-inflammatory activity of lipophilic epigallocatechin gallate (EGCG) derivatives in LPS-stimulated murine macrophages. Food Chem 134: 742–748. 10.1016/j.foodchem.2012.02.172 23107686

[44] Cavet ME, Harrington KL, Vollmer TR, Ward KW, Zhang JZ (2011) Anti-inflammatory and anti-oxidative effects of the green tea polyphenol epigallocatechin gallate in human corneal epithelial cells. Mol Vis 17: 533–542. 21364905 PMC3044696

[45] Zhou P, Yu JF, Zhao CG, Sui FX, Teng X, et al (2013) Therapeutic potential of EGCG on acute renal damage in a rat model of obstructive nephropathy. Mol Med Rep 7: 1096–1102. 10.3892/mmr.2013.1296 23358654

[46] Wang Y, Wang B, Du F, Su X, Sun G, et al (2015) Epigallocatechin-3-Gallate Attenuates Oxidative Stress and Inflammation in Obstructive Nephropathy via NF-kappaB and Nrf2/HO-1 Signalling Pathway Regulation. Basic Clin Pharmacol Toxicol. 10.1111/bcpt.12383 25625183

[47] Wei Q, Dong G, Yang T, Megyesi J, Price PM, et al (2007) Activation and involvement of p53 in cisplatin-induced nephrotoxicity. Am J Physiol Renal Physiol 293: F1282–1291. 17670903 10.1152/ajprenal.00230.2007 PMC2792752

[48] Zhou H, Kato A, Yasuda H, Miyaji T, Fujigaki Y, et al (2004) The induction of cell cycle regulatory and DNA repair proteins in cisplatin-induced acute renal failure. Toxicol Appl Pharmacol 200: 111–120. 15476864 10.1016/j.taap.2004.04.003

[49] Nasri H, Ahmadi A, Baradaran A, Nasri P, Hajian S, et al (2014) A biochemical study on ameliorative effect of green tea (Camellia sinensis) extract against contrast media induced acute kidney injury. J Renal Inj Prev 3: 47–49. 10.12861/jrip.2014.16 25340167 PMC4206045

[50] Pan H, Mukhopadhyay P, Rajesh M, Patel V, Mukhopadhyay B, et al (2009) Cannabidiol attenuates cisplatin-induced nephrotoxicity by decreasing oxidative/nitrosative stress, inflammation, and cell death. J Pharmacol Exp Ther 328: 708–714. 10.1124/jpet.108.147181 19074681 PMC2682269

[51] Mukhopadhyay P, Pan H, Rajesh M, Batkai S, Patel V, et al (2010) CB1 cannabinoid receptors promote oxidative/nitrosative stress, inflammation and cell death in a murine nephropathy model. Br J Pharmacol 160: 657–668. 10.1111/j.1476-5381.2010.00769.x 20590569 PMC2931565

[52] Yao X, Panichpisal K, Kurtzman N, Nugent K (2007) Cisplatin nephrotoxicity: a review. Am J Med Sci 334: 115–124. 17700201 10.1097/MAJ.0b013e31812dfe1e

[53] Ali BH, Al Moundhri MS (2006) Agents ameliorating or augmenting the nephrotoxicity of cisplatin and other platinum compounds: a review of some recent research. Food Chem Toxicol 44: 1173–1183. 16530908 10.1016/j.fct.2006.01.013

[54] Daugaard G, Abildgaard U (1989) Cisplatin nephrotoxicity. A review. Cancer Chemother Pharmacol 25: 1–9. 2686850 10.1007/BF00694330

[55] Waseem M, Kaushik P, Parvez S (2013) Mitochondria-mediated mitigatory role of curcumin in cisplatin-induced nephrotoxicity. Cell Biochem Funct 31: 678–684. 10.1002/cbf.2955 23408677

[56] Santos NA, Bezerra CS, Martins NM, Curti C, Bianchi ML, et al (2008) Hydroxyl radical scavenger ameliorates cisplatin-induced nephrotoxicity by preventing oxidative stress, redox state unbalance, impairment of energetic metabolism and apoptosis in rat kidney mitochondria. Cancer Chemother Pharmacol 61: 145–155. 17396264 10.1007/s00280-007-0459-y

[57] Santos NA, Catao CS, Martins NM, Curti C, Bianchi ML, et al (2007) Cisplatin-induced nephrotoxicity is associated with oxidative stress, redox state unbalance, impairment of energetic metabolism and apoptosis in rat kidney mitochondria. Arch Toxicol 81: 495–504. 17216432 10.1007/s00204-006-0173-2

[58] Green DR, Reed JC (1998) Mitochondria and apoptosis. Science 281: 1309–1312. 9721092 10.1126/science.281.5381.1309

[59] Meng Q, Velalar CN, Ruan R (2008) Effects of epigallocatechin-3-gallate on mitochondrial integrity and antioxidative enzyme activity in the aging process of human fibroblast. Free Radic Biol Med 44: 1032–1041. 10.1016/j.freeradbiomed.2007.11.023 18206666

[60] Ahn WS, Huh SW, Bae SM, Lee IP, Lee JM, et al (2003) A major constituent of green tea, EGCG, inhibits the growth of a human cervical cancer cell line, CaSki cells, through apoptosis, G(1) arrest, and regulation of gene expression. DNA Cell Biol 22: 217–224. 12804120 10.1089/104454903321655846

[61] Albrecht DS, Clubbs EA, Ferruzzi M, Bomser JA (2008) Epigallocatechin-3-gallate (EGCG) inhibits PC-3 prostate cancer cell proliferation via MEK-independent ERK1/2 activation. Chem Biol Interact 171: 89–95. 17931610 10.1016/j.cbi.2007.09.001

[62] Braicu C, Gherman CD, Irimie A, Berindan-Neagoe I (2013) Epigallocatechin-3-Gallate (EGCG) inhibits cell proliferation and migratory behaviour of triple negative breast cancer cells. J Nanosci Nanotechnol 13: 632–637. 23646788 10.1166/jnn.2013.6882

[63] Chen D, Wan SB, Yang H, Yuan J, Chan TH, et al (2011) EGCG, green tea polyphenols and their synthetic analogs and prodrugs for human cancer prevention and treatment. Adv Clin Chem 53: 155–177. 21404918 10.1016/b978-0-12-385855-9.00007-2 PMC3304302

[64] Lecumberri E, Dupertuis YM, Miralbell R, Pichard C (2013) Green tea polyphenol epigallocatechin-3-gallate (EGCG) as adjuvant in cancer therapy. Clin Nutr 32: 894–903. 10.1016/j.clnu.2013.03.008 23582951

